# Iodine and Its Preparations in the Therapeutics of Infancy

**Published:** 1877-04-20

**Authors:** 


IODINE AND ITS PREPARATIONS IN THE THERAPEUTICS OF INFANCY.—In a clinical lecture delivered at the Paris Hospital for Children, M. Jules Simon lays particular stress upon the following points:—Tincture of iodine should not be applied pure in tubercular children; it should be diluted with either glycerine or some unguent. Neither iodide or potassium nor iodide of iron should be given to children under two years of age, except perhaps in cases of acute hereditary syphilis, where small doses may be administered. It may be given to the nurse if the child has not been weaned. Older children bear the drug well.—[Boston Med. Journal.

